# Development of a Kidney Microphysiological System Hardware Platform for Microgravity Studies

**DOI:** 10.21203/rs.3.rs-3750478/v1

**Published:** 2023-12-22

**Authors:** Catherine Yeung, Kendan Jones-Isaac, Kevin Lindberg, Jade Yang, Jacelyn Bain, Micaela Ruiz, Greta Koenig, Paul Koenig, Stefanie Countryman, Jonathan Himmelfarb, Edward Kelly

**Affiliations:** University of Washington; University of Washington; University of Washington; University of Washington; University of Washington; University of Washington; BioServe Space Technologies; BioServe Space Technologies; BioServe Technologies; Kidney Research Institute; Univ of Washington

**Keywords:** Kidney, Microphysiologic Systems, Microgravity, International Space Station, Tissue Chips in Space

## Abstract

Study of the physiological effects of microgravity on humans is limited to non-invasive testing of astronauts. Microphysiological models of human organs recapitulate many functions and disease states. Here we describe the development of an advanced, semi-autonomous hardware platform to support kidney microphysiological model experiments in microgravity.

## Introduction

The microgravity environment induces a plethora of pathophysiological changes that resemble accelerated aging including wasting of skeletal muscle,^1^ bone demineralization^2^, and metabolic and cardiovascular dysregulation ^3, 4,5^. In the case of bone mineral homeostasis, the kidneys control the excretion and retention of calcium, phosphate and other essential ions^6^. The kidneys are also responsible for generation of the active form of vitamin D, 1α,25-(OH)_2_ vitamin D_3_, which plays a critical role in a multitude of biological functions including bone health^7^.

Directly evaluating the impact of microgravity on kidney function at the molecular and cellular level is obviously not feasible in astronauts due to the invasiveness and inherent risk of performing renal biopsies^8^. While studies can be conducted in rodents, the results may not truly reflect changes occurring in humans. To address the question of how microgravity affects human physiology, the National Center for Advancing Translational Sciences (NCATS) at the National Institutes of Health formed the “Tissue Chips in Space” program (Tissue Chips in Space | National Center for Advancing Translational Sciences (nih.gov)) that leveraged novel tissue engineering platforms to recapitulate human physiology in the environment of space. Selected research teams were allowed to each send two projects to the International Space Station National Lab (ISSNL) during the four-year funding period.

Microfluidic-based microphysiologic systems (MPS) represent an advancement in cell culture techniques aimed at better replicating the tissue-specific *in vivo* environment. We have previously reported our development of a MPS-based model of the kidney proximal tubule (PT-MPS) utilizing a commercially available platform developed by Nortis Inc.^9^ The Nortis^™^ system is designed for use with a tubing-free pneumatic-driven pump system but requires a substantial footprint, presents logistical challenges within the lab and is not suitable in all research contexts. Therefore, the Kidney Chip Perfusion Platform (KCPP), a piston-based device, was developed by BioServe Space Technologies to support an MPS-based kidney proximal tubule model.

The PT-MPS has been used to study a variety of disease states (e.g., aristolochic acid nephropathy and proteinuria^10,11^) and the responses to drug/xenobiotic-induced kidney injury^12–14^. In addition, the robustness of this system was independently tested in collaboration with the NCATS-funded Tissue Chips Testing Centers^15,16^. To test the premise that microgravity is an accelerated environment for aging/disease progression, we evaluated proteinuria, kidney vitamin D metabolism, and nephrolithiasis (kidney stone disease)^17^. To adapt the system to the infrastructure aboard the ISSNL, we created a completely novel hardware support system with BioServe Space Technologies, our Implementation Partner and Payload Developer. BioServe Space Technologies is a research center within the University of Colorado, Boulder and has a proven track record designing life sciences hardware for microgravity experiments with their hardware having flown on over 85 space flight missions. Herein we report the development of the KCPP in support of two missions to the ISSNL.

## Results

### Kidney Chip Perfusion Platform System Overview

**Figure 1:**
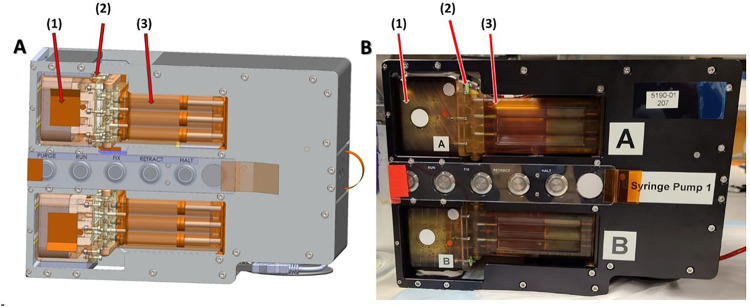
Schematic (A) and real life (B) view of the novel KCPP programmable perfusion platform designed by BioServe Space Technologies showing six Triplex chips (1) situated within a housing unit after integration into the adapter unit (2) and media cassette (3).

In partnership with BioServe Space Technologies, we developed the KCPP hardware, addressed NASA safety and regulatory requirements, and facilitated the transition to a spaceflight certified and capable system. The KCPP is a precision, syringe pump-based platform designed to perfuse up to six Nortis^™^ Triplex (each unit has three independently perfused tubules) PT-MPS built to support the NIH/NCATS Kidney Cell experiments. The platform is composed of five components, the kidney MPS, the MPS housing and valve block, media cassettes, fixative cassettes, and the programable precision syringe pump. Each KCPP as shown in Fig. 1 is comprised of over 2500 custom-designed and machined components. In the lab, preparing and assembling these components for experiments is a lengthy process and requires sustained, active engagement. The astronauts aboard the ISS have a set number of hours to operate scientific experiments and operate on a strict schedule. The innovation of the KCPP over the in-lab process is a dramatic reduction in complexity and time commitment. For example, in the lab, switching between maintenance and experimental media can be a multi-hour effort. This process was simplified with a pump interfacing to the MPS housing and valve block which can accept pre-loaded media or fixative cassettes. The pump provides a continuous flow of media or fixative while maintaining temperature control at 37° C. The pump uses a stepper motor to provide translation of a carriage which simultaneously depresses 18 syringe plungers. Preloading the media and fixative cassettes on the ground during the final pre-launch preparation phase streamlines the on-orbit protocol followed by the assigned astronaut on board the ISSNL. Additionally, the software for the pump only requires 5 operating modes for the experiment: the “Purge” command initiates the pump to engage the syringe pistons an prime the channels connecting the cassettes and the MPS, the “Run” command initiates perfusion with media at 0.5 μL/min, the “Fix” command perfuses fixative at 10 μL/min, the “Retract” command resets the pump plunger positions for sample housing and valve block and media/fixative cassette removal, and “Halt” stops all piston movement. The perfusion rate for media and fixation is programmable. Thus, while the KCPP is a complex work of engineering, the interface for users on the ISSNL is intuitive and user-friendly.

Media is loaded into nine channels separated by effluent bag cavities within one media cassette. The media is contained in the channels between an O-ring piston and a septum. A cannula from the valve block pierces the septa when installed and allows the piston to push media into the PT-MPS. The media flows through the PT-MPS and is collected in the effluent bags that are sealed with septa that are also pierced by cannula. The effluent bag cavities have containment plugs with O-rings on a retention plate. The waste media fills the effluent bags and is contained for post-flight analysis.

### Chip Housing & Valve Block

**Figure 2:**
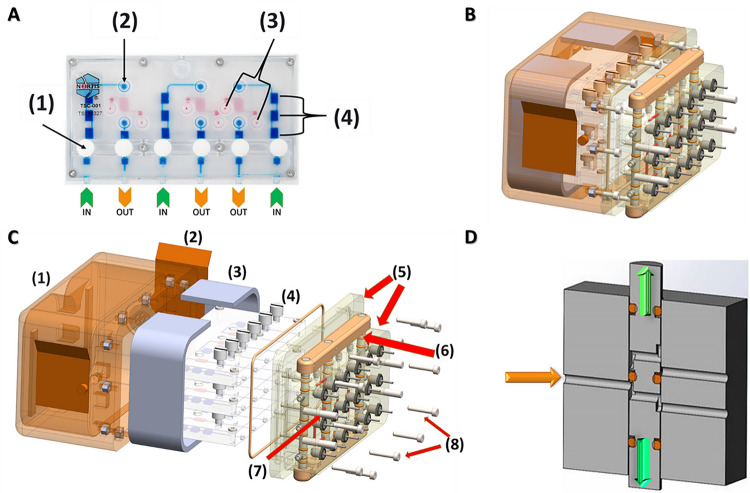
Nortis TCC, Triplex Housing & Valve Block Assembly ***(A)*** Schematic depicting a Nortis Triplex Chip including; (1) port valve, (2) injection port, (3) matrix plug holes, (4) bubble traps, ***(B)*** An integrated view of a Chip Housing and Valve Block Assembly, ***(C)*** An exploded view of a Chip Housing & Valve Block Assembly including; (1) Triplex Housing, (2) Vent Cover, (3) Absorbent Padding, (4) Nortis Triplex Chips, (5) Valve Block, (6) Valve Bar, (7) Cannula, (8) Alignment Pins, ***(D)*** Diagram of the valve block assembly oriented such that media flows from left to right depicting the internal mechanism set to the purge configuration.

The MPS housing and valve block system is a protective sealed enclosure, designed with functions for purging bubbles during media or fixative cassette installation (Fig. 2). Considerable effort is taken in the lab to mitigate the risk of bubbles entering the MPS since this will lead to disruption of media flow and compromise the integrity of the PTEC lumen. Because of the unpredictable nature of air bubbles in microgravity, they may not be subject to the same effects of buoyancy as on earth. Thus, it is possible that bubbles may bypass the traps in the MPS that are designed to utilize that buoyancy to trap bubbles above the path of the media. The enclosure interfaces to the media cassette and fixative cassette via four alignment pins and 18 cannulas. The housing vents are sealed with two adhesive covers during launch operations to maintain a 5% CO_2_ and 100% humidity environment within the MPS housing. The valve block is designed with a valve bar system to direct flow through the valve block. Purging is performed when the valve bar is in the upper position as shown in Fig. 2D. When the valve bar is in purge mode, flow is diverted from the PT-MPS directly into the effluent bags. When the valve bar is in flow mode, flow is directed into the PT-MPS.

### Media and Fixative Cassettes

**Figure 3:**
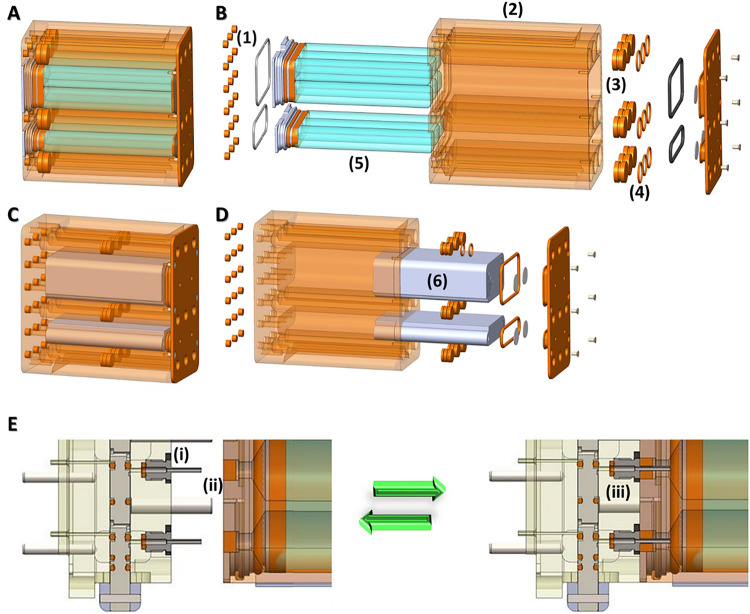
Media & Fixative Cassettes, ***(A)*** Integrated view of a media cassette assembly, ***(B)*** Exploded view of a media cassette assembly, ***(C)*** Integrated view of a fixative cassette assembly, ***(D)*** Exploded view of a media cassette assembly (1) septa, (2) polyetherimide block, (3) piston, (4) Piston o-ring, (5) FEP bags (media cassette only), (6) absorbent padding (fixative cassette only), **(E)** Diagram depicting the self-sealing septa mechanism in separated **(L)** and connected **(R)** configurations.

The media cassette was designed to integrate directly with the chip housing and valve block and the KCPP to provide sufficient media to perfuse the PT-MPS for 10 days at a rate of 750 μL/day (Fig. 3A). The cassette consists of nine individual channels machined into an Ultem thermoplastic resin block. Each channel has a usable volume of 7.75 mL. The fluid is dispensed by mechanical plunger translation via the syringe pump. Once the fluid has passed out of the cassette and through the PT-MPS it returns to the housing and is stored in individually sealed bags in an adjacent chamber to the media channels. The fluid interfaces with the valve block via 18 cannulas piercing the corresponding septa in the bottom of the media cassette.

The sample effluent collection volume is sealed at the top of the cassette which provides an additional level of containment. When two levels of containment are required during cassette change out operations, the KCPP system can be operated within the Microgravity Science Glovebox (MSG) or Life Science Glovebox (LSG) which provide an additional level of containment during astronaut manipulations of the media or fixative cassettes or the KCPP in general.

The fixative cassette is a modified version of the media cassette (Fig. 3B). The cassette provides fixative for the final stage of the experiment to preserve the cells in the PT-MPS. The cassette has nine individual channels machined into a block of Ultem. Each channel has a maximum volume of 3.8 ml. The fluid is dispensed via mechanical plunger translation via the syringe pump. Once the fluid has passed out of the cassette and through the PT-MPS it returns to the housing and is absorbed into layers of absorbent material in adjacent chambers to the fixative channels.

The fluid interface to the valve block is via 18 cannulas piercing the corresponding septa in the bottom of the fixative cassette. The fixative cassette provides two levels of containment using O-rings on the pistons. Additional containment can be provided via outer bags, if needed.

### KCPP Integration

**Figure 4:**
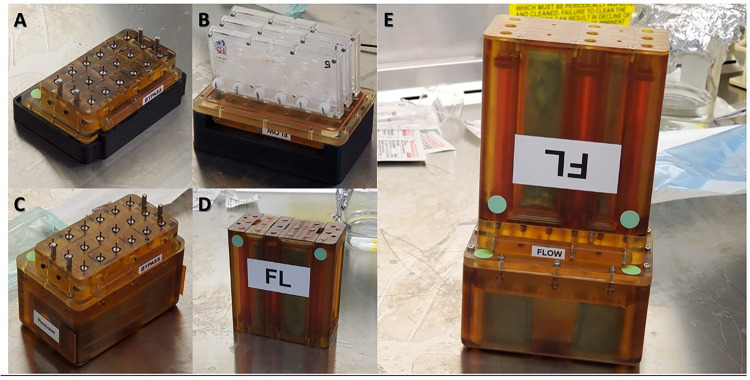
Stepwise integration of the chip environment, ***(A)*** Valve block, ***(B)*** Three triplex chips integrated onto the valve block, ***(C)*** Valve block & triplex housing integrated assembly, ***(D)*** Launch media cassette, ***(E)*** Media cassette, valve block & triplex housing.

The integration and assembly of the individual components of the KCPP are shown in Fig. 4. In brief, Figs. 4A–C depicts a valve block, PT-MPS and integrated assembly, respectively. A media cassette is shown in and all the assembled components are seen in Fig. 4E.

### KCPP Space Reduction Advancements

**Figure 5:**
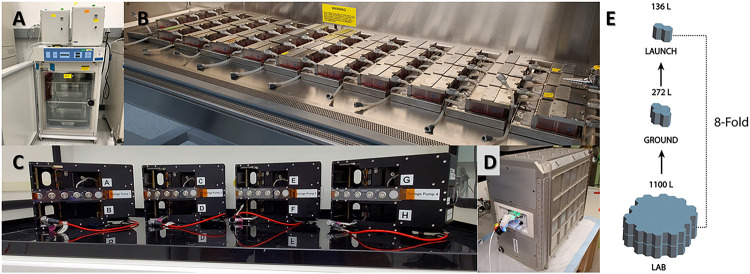
Reduction of the chip experimental footprint. ***(A)*** Image depicting the amount of space 24 Triplex Chips occupy when the platforms and shelving is placed with the docking stations within tissue culture incubators, with the pneumatic pumps mounted on the top of the incubator, ***(B)*** Image depicting the amount of laboratory space 44 Nortis Triplex Chips occupy when attached to platforms and shelves in a six foot biosafety cabinet, ***(C)*** Image of KCPP programmable perfusion platforms designed by BioServe Space Technologies Four platforms are capable of perfusing 24 Nortis Triplex Chips, ***(D)*** The powered locker used in the Kidney Chips experiment supplies power to four KCPP pumps, ***(E)*** The KCPP platform reduces the space required to perfuse 48 Nortis Triplex Chips by 8-fold enabling astronauts to work within the limited space of the Life Sciences Glovebox. [2X incubators + 1X CO_2_ Tank = 1100 L Each Locker measures ~68 L (67.3L)]

Although the overall footprint of an individual PT-MPS in the lab is small, the specialized equipment required to perfuse the devices is relatively large. As shown in Fig. 5A/B, the individual components required to run experiments in our conventional fashion require an entire tissue culture incubator. The availability of space on ISSNL is limited but the KCPP reduces that required footprint 8-fold (1100 L to 136 L) allowing 24 PT-MPS to be housed and perfused within the locker space allocated to our group on board the ISSNL (Figs. 5C–E). As previously stated, the Nortis^™^ pneumatic system does not require the use of tubing but the BioServe platform is syringe pump-based. We have previously used commercially available syringe pumps to run PT-MPS experiments and 24 PT-MPS require eight of these pumps to independently perfuse each of the 72 PT-MPS tubules. As shown in **Supplementary Fig. 1,**, the system accommodates two pumps per tissue culture incubator, necessitating four separate incubators for 24 PT-MPS. In addition to the significant space reductions from the KCPP, we have also eliminated the use of tubing, as the PT-MPS directly interface with the media blocks in the valve assembly. With syringe pumps, each individual PT-MPS tubule requires approximately 1 meter of tubing to connect media syringes outside of the incubators with PT-MPS within the incubator (**Suppl. Figure 1**). Thus, in addition to creating a simplistic user-interface for operation on the ISSNL, the KCPP exponentially shrinks the footprint requirements compared to conventional terrestrial PT-MPS experiments.

### Testing and Validation of the System

**Figure 6:**
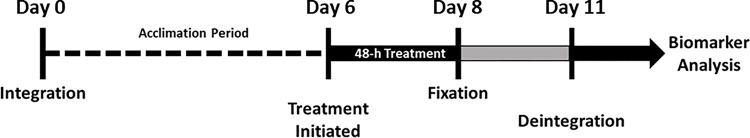
Experimental timeline for engineering validation test

An experiment validation test (EVT) was performed prior to launch to assess the ability of the perfusion platform to maintain kidney PT-MPS cultures over the duration of the proposed experiments (Fig. 6). Kidney PT-MPS were loaded into the MPS housing and then integrated with the valve block and then into the perfusion platform. The devices were then cultured for six days in maintenance media to simulate a period of acclimation to microgravity. At day six, maintenance media cassettes were exchanged for treatment media cassettes and perfusion was continued for a 48-h treatment phase. At day eight, treatment media cassettes were removed and exchanged for fixative cassettes containing either RNAlater^®^ or formalin. The effluent from both the maintenance and treatment media were stored at −80°C for later analysis. Once the fixative cassette was integrated with the system, fixative/preservative was perfused for 1 hour after which the platform components were deintegrated and the PT-MPS were stored at −80° C or 4° C for later analysis.

**Figure 7:**
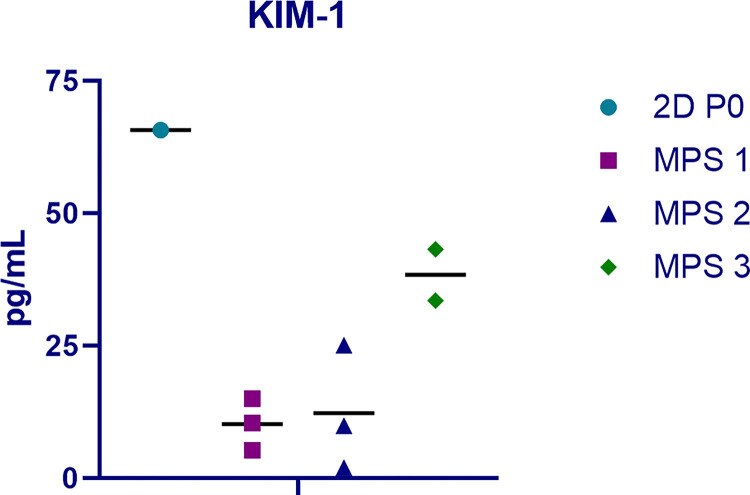
KIM-1 levels in effluents of kidney MPS cultured for 11 days in the KCPP and in 2D cultured PTECs at passage 0 after 7 days.

Kidney Injury Molecule-1 (KIM-1) is a protein secreted into the urinary filtrate by proximal tubule health of our PT-MPS during the EVT, we measured the secretion of KIM-1 in effluents. We have previously shown that basal secretion of KIM-1 by PT-MPS is low but is markedly increased in response to nephrotoxic insults^14–16^. As shown in Fig. 7, we observed low levels of KIM-1 from multiple PT-MPS evaluated in the EVT. For reference, a sample of 2D PTEC culture supernatant was included, but it should be noted that higher KIM-1 levels are expected in 2D cultures due to the cells being in a proliferative state while PTECs cultured in MPS devices are not proliferating^15^.

### KCPP System Performance

**Figure 8:**
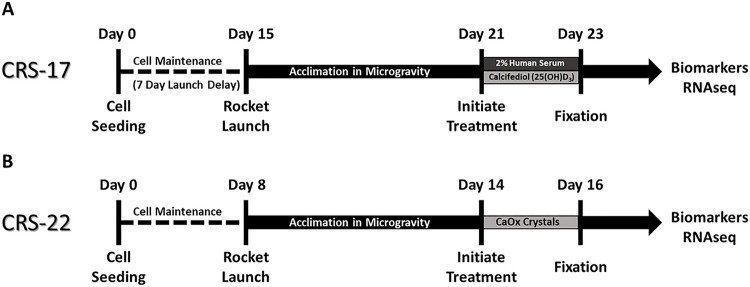
Experimental timelines for: ***(A)*** CRS-17 & ***(B)*** CRS-22

To date, we have completed two launches of the KCPP system to the ISSNL. The first launched on board SpaceX Commercial Resupply Services mission 17 (CRS-17) and the second on SpaceX CRS-22. On the first launch we evaluated vitamin D metabolism and proteinuric responses and the second launch on CRS-22 studied a calcium oxalate microcrystal model of nephrolithiasis. To assess overall performance of the KCPP hardware, we evaluated the ability to recover PT-MPS effluents for biomarker analyses as well as successful perfusion of RNAlater^™^ for gene expression studies. The basic study design and timelines for CRS-17 and CRS-22 are shown in Figs. 8A/B, respectively. Each launch consisted of 24 PT-MPS in-flight (microgravity) with a matched cohort of 24 ground-based PT-MPS. The CRS-17 launch consisted of 4 different PTEC donors (two males & two females) while CRS-22 included 6 different donors (three males & three females). The number of samples obtained for RNAseq analysis are shown in Tables 1 and 2 for CRS-17 and -22, respectively while Tables 3 and 4 show a similar breakdown for effluent retrievals for CRS-17 and -22, respectively.

The criteria for determining a “usable” sample for RNAseq was based on the ability to retrieve RNA from the PT-MPS tubules with a detergent solution, and subsequent total RNA isolation. Quality controls included Bioanalyzer^™^ RNA integrity determination, RNA concentrations as well as subsequent RNAseq analysis (data not shown) and reported in **Table 5.** The criterium for “usable” sample for effluent analysis was based on retrieval of media in individual effluent bags after thawing of the KCPP media cassette blocks. It is worth noting the differences in the rates of “usable samples” between CRS-17 and CRS-22. In CRS-17, approximately 30% of the samples (RNAseq and effluents) were unusable for both flight and ground due to mold contamination of the PT-MPS. In contrast, nearly 100% of the samples were usable in CRS-22. The mold contamination observed in CRS-17 was not related to KCPP performance. Instead, it was likely driven by a combination of multiple launch delays that necessitated greater handling/transport of the PT-MPS from standard cell culture incubators to launch lockers and small amounts of residual media on the cell injection port on the PT-MPS. Approximately one week into the launch delay, an additional media cassette exchange procedure was carried out to ensure a fresh supply of media to the PT-MPS devices. To mitigate these issues for CRS-22, we employed PT-MPS cleaning protocols as well as applied a medical-grade silicone-based sealant (Silastic A^®^) over the PT-MPS cell injection ports. It is also worth noting that the issues with launch delays in CRS-17 did not occur with CRS-22.

## Discussion

KCPP is an integrated, automated, piston-based perfusion platform and enclosed cell culture environment designed to support MPS-based life sciences experimentation on board the ISSNL. Its compact design enables a significant reduction in the logistical challenges and spatial footprint required to implement these experiments aboard the confined space of the ISSNL. KCPP has been verified and space flight certified and is compliant with current NASA safety and interface requirements for space flight and use aboard the ISSNL. The system has been successfully utilized to support two space-based experimental studies designed to test the impact of microgravity on the function and pathophysiology of PTECs cultured in MPS aboard the ISSNL. In each instance, KCPP performed nominally and facilitated the execution of experiments otherwise impossible to be conducted terrestrially in simulated microgravity. The improvement in of samples recovery between missions emphasizes the importance of developing countermeasures against factors responsible for tubule or MPS device attrition. Looking to the future, extended studies using the KCPP system will facilitate the understanding of the long-term effects of spaceflight on renal physiology. Future development of autonomous MPS-based platforms can be used to predict human health concerns caused by spaceflight and long-term residence in microgravity that will occur during long term human space exploration.

## Methods

### Tissue Acquisition & Cell Culture:

Whole human kidneys that were not suited for human transplantation were obtained from Novabiosis, Inc. (Research Triangle Park, NC) with all patient identifiers removed in accordance with a biospecimens procurement agreement. Primary human proximal tubule epithelial cells (PTECs) were isolated by mechanical and enzymatic dissociation and cultured as previously reported^18^. PTEC cultures were maintained serum-free in DMEM/F12 (Gibco, Grand Island, NY, Cat. # 11330-032) supplemented with 1× insulin-transferrin-selenium-sodium pyruvate (ITS-A, Gibco, Cat. # 51300044), 50 nM hydrocortisone (Sigma, St. Louis, MO, Cat. # H6909), and 1× Antibiotic-Antimycotic (Gibco, Cat. # 15240062). Upon reaching 75–80% confluence, PTECs were passaged by enzymatic digestion with 0.05% trypsin EDTA (Gibco, Cat. # 25200056) and manual cell scraping to obtain a single-cell suspension which was subsequently neutralized with defined trypsin inhibitor/DTI (Gibco, Cat. # R007100) at a volume:volume ratio of 2:1 DTI:trypsin, then the cells were pelleted by centrifugation at 200 × g for 7 minutes, resuspended in maintenance media, and plated in cell culture treated flasks at > 25% confluency (referred to as passage 1 or P1). For both EVTs and CRS-17/22 missions, media cassettes were loaded and then stored for 1 week at 4° C before being warmed to 37° C immediately prior to integration with the KCPP. At the end of the treatment duration, sample effluents were frozen at −80° C.

#### Preparation of Nortis Kidney MPS

Kidney MPS devices were purchased from Nortis, Inc (Woodinville, WA). Device preparation and PTEC injections were performed by the investigators as previously reported^9^. PTEC MPS cultures were maintained serum-free in DMEM/F12 (Gibco, 11330-032) supplemented with 1× insulin-transferrin-selenium-sodium pyruvate (ITS-A, Gibco, 51300044), 50 nM hydrocortisone (Sigma, H6909), and 1x Antibiotic-Antimycotic (Gibco, 15240062). In brief, for all the experiments run for experimental validation testing (EVT) as well as for Commercial Resupply Services (CRS) missions CRS-17 and CRS-22, PTECs of passage 2 or lower were used from each individual donor kidney. In the cases of experiments run for CRS-17 & CRS-22, PTECs were shipped on dry ice to a lab at Kennedy Space Center. Following recovery from cryopreservation and expansion in 2D culture, MPS were seeded and allowed to culture as detailed in Figs. 6 and 8. As part of the EVT experimental design, a media cassette change was performed eight days after initiating KCPP flow.

#### Quantification of organ-specific injury biomarker KIM-1

DuoSet^©^ ELISA kits were used to quantify human KIM-1 (R&D Systems, Minneapolis, MN, Cat. # DY1780B) in PT-MPS effluents following the manufacturer’s protocol. In brief 50–100 μL of effluent were tested in duplicates and concentrations determined based on the standard curves generated from manufacturer-supplied controls.

#### RNAseq data generation and analysis

To collect RNA samples from PTEC tubules, the PT-MPS devices were flushed with a volume of 1 mL RLT buffer (Qiagen, #79216) delivered through the abluminal inlet using a 1 mL slip-tip syringe (BD, 309659) equipped with a 22-gauge needle (BD, 305142) and collected at the outlet port. The RNA samples in RLT buffer were stored at −80° C until extraction which was performed as described by Lidberg et. al ^19^.
